# Intestinal tract and parenteral multi-organ sequential pathological injury caused by necrotizing enterocolitis

**DOI:** 10.1186/s12887-020-02304-5

**Published:** 2020-09-02

**Authors:** Fu-Sheng Wang, Meng-Lu Yu, Wei-Zhong Li, Kai Hong, Chen-Bin Xu, Guang-Huan Wang

**Affiliations:** 1 Department of Pediatric Surgery, The Second Affiliated Hospital of Shantou University Medical College, Shantou, 515041 China; 2 Department of Neonatal, The Second Affiliated Hospital of Shantou University Medical College, Shantou, China; 3 Department of Clinical Laboratory, The Second Affiliated Hospital of Shantou University Medical College, Shantou, China

**Keywords:** Necrotizing enterocolitis, Bcl2-related X gene, Platelet-activating factor, Proliferating cell nuclear antigen

## Abstract

**Background:**

To explore the relationship between the pathological changes of the colon, terminal ileum, lung, liver and kidney, and the changes of Bax, PCNA and PAF in a rat model of NEC.

**Methods:**

One hundred and forty neonatal SD rats were randomly divided into NEC group and control group (70 in each group). NEC group was given hypoxia, cold stimulation and artificial feeding twice a day for 3 consecutive days. The control group was only fed normally. After modeling, From the 1st day to the 7th day, 10 rats were sampled in each group for pathological examination of colon, terminal ileum, lung, liver and kidney tissue. The levels of Bax, PCNA and PAF were investigated by immunohistochemistry.

**Results:**

Compared with the normal group, in the NEC group, on the 1st day, the colon, terminal ileum, lung, liver and kidney showed inflammatory damage. On the 5th day, the inflammatory injury was reduced. The inflammation disappeared on the 7th day. There were differences in the time of apoptosis in the intestine. In the intestine, the proliferation of PCNA was weak at first and then strong. Bax in liver and kidney showed marked apoptosis and apoptosis time increased in the lung. The expression of PCNA increased in lung, liver and kidney, and the expression of PAF increased in lung and liver.

**Conclusions:**

NEC can lead to secondary injury of different degrees in colon, terminal ileum, lung, liver and kidney, and the degree and time of injury and repair were different. In general, organ repair played a leading role on the 4th day after modeling.

## Background

Neonatal NEC is a serious life-threatening gastrointestinal emergency in the neonatal stage. It is one of the most destructive diseases in neonates [1]. Research on the pathogenesis and specific treatment of NEC has become an essential subject in pediatrics. At present, the study of NEC mainly focuses on the aetiology and pathogenesis, as well as the protection and prevention of injured intestinal organs. However, there is a lack of systematic research and understanding of the pathological and functional changes of various organs in the whole body after the occurrence of NEC. We established an animal model of NEC to study pathological changes of the colon, terminal ileum, lung, liver and kidney after induced NEC, and then detected the expression of Bax, PCNA and PAF in these organs [2].

Bax is a vital apoptosis promoting gene, and its rise indicates that the cell starts the apoptosis process. PCNA plays an important role in the initiation of cell proliferation and is a good indicator of cell proliferation. PAF is a phospholipid medium induced by endotoxin and cytokines. It can improve vascular permeability, promote platelet aggregation, enhance the release of arachidonic acid and have a negative inotropic effect on the heart. In this study, the above three cytokines were detected in SD rats. Human beings, like SD rats, produce these cytokines when they are exposed to NEC [3–5]. We hope to study the pathological progress and the change rule of the outcome of NEC from three aspects of apoptosis, proliferation and inflammatory damage.

## Methods

### Materials

The study protocol was approved by the Medical Animal Care and Welfare Committee of Shantou University Medical College. SD rats (grade: SPF) were from the Shantou University Medical College Laboratory Animal Center. The rats were aged 6–8 weeks (weight: 205-298 g), which male and female 1:2 caged. They were provided with clean drinking water and full price feed. We anesthetized the rats before taking samples or causing pain in rats. The rats were killed by CO2 suffocation after the experiment. The newborn rats born from these SD rats were used as experimental animals (weight: 6-7 g), all of the rats were healthy.

One hundred and forty rats were randomly divided into NEC group and control group, 70 rats in each. Their average weight is 6.7 g, and their gender is random. A rat model for NEC was established by artificial feeding of dairy substitutes, hypoxia and cold stimulation (100% nitrogen hypoxia 90 s, 4 °C cold stimulation 10 min, twice a day for 3 consecutive days) [6–8]. In the NEC group, the induction of NEC was initiated on the 3rd day after birth. The day-age of the rats in the control group is the same as that of the rats in the corresponding NEC group.

### Sampling

On the 1st, 2nd, 3rd, 4th, 5th, 6th and 7th day after modeling, 10 rats were randomly euthanized by cervical dislocation. The abdominal cavity was opened with hemostatic forceps and surgical scissors to separate the colon, terminal ileum, lung, liver and kidney. Images were taken with a digital camera (MVC-FD 91, Sony).

### Pathological section preparation and hematoxylin-eosin staining

The above specimens were fixed in 10% formalin for 48 h after rinsing with normal saline, then embedded in paraffin. Tissue sections of 4-6 μm thickness were prepared and stained with HE. Criteria: After hematoxylin-eosin staining, the morphological changes of intestinal tissue were observed under light microscopy. The intestinal tissue injury score was divided into 4 grades - 0: normal; 1: slight separation of submucosa and / or lamina propria; 2: moderate separation of submucosa and / or lamina propria, or oedema of submucosa and muscularis; 3: severe separation of submucosa and / or lamina propria, and (or) severe oedema of submucous and muscular layer, local villi shedding; 4: disappearance of intestinal villi with intestinal necrosis. A histological score ≥ 2 was defined as NEC.

### Immunohistochemical staining

Daily, from the 1st through 7th day after modeling, the expression of Bax, PCNA and PAF in the colon, terminal ileum, lung, liver and kidney of the NEC and control groups were observed. Judgment criteria: the staining reaction was observed under an optical microscope, and positive staining was defined as a brownish-yellow particle deposition in the nucleus or cytoplasm. Image-Pro Plus v5.1 image analysis software was used for analysis.

### Statistical analysis

The experimental results were analyzed by SPSS 21.0 software. The measurement data were expressed by “mean ± standard deviation”, and the difference was statistically significant when *P* < 0.05. ANOVA was used for comparison among groups. If *P* < 0.05, t-test was used (every two groups were compared at the same time). ANOVA was used to compare multiple times in the same group. If *P* < 0.05, LSD test within the group was performed (the same group was compared at different time points). The analysis of the time trend is based on the results of LSD test.

## Results

### Statistical results

At the same time, there was a significant difference in cytokine production between the two groups (F = 40.332, *P* = 0.001). The test results of cytokines expression in different organs of the NEC group and control group were as follows (Table 1). Except for PAF expressed in the liver, there was a significant difference in other control groups (*P* < 0.05).

**Table 1:** T-test results (t, *P-value*) of cytokines expressed in organs of the NEC group and the control group on different days

	colon	ileum	lung	liver	kidney
Bax	3.424,0.005	3.977,0.002	3.953,0.002	2.588,0.024	2.757,0.023
PCNA	−3.578,0.004	−4.075,0.002	3.580,0.040	4.692,0.002	5.461,0.000
PAF	5.246,0.000	4.474,0.001	−2.835,0.015	1.599,0.136	−2.329,0.044

There was no significant difference in Bax, PCNA and PAF in the three control groups at different days (*P* = 0.459, 1.000, 0.923, respectively). In NEC group, the difference of Bax mean from day 1 to day 7 in each organ was statistically significant (F = 3.154, *P* = 0.017), the difference of PCNA mean from day 1 to day 7 in each organ was statistically significant (F = 5.141, *P* = 0.001), the difference of PAF mean from day 1 to day 7 in colon and ileum was statistically significant (F = 4.300, *P* = 0.039), but the difference of PAF mean from day 1 to day 7 in lung, liver and kidney was not statistically significant (F = 2.080, *P* = 0.121). For the statistical significance of the mean value of each tissue of each group, we used LSD to test them (Table 2). The test results were used for time trend analysis.

**Table 2:** Results of cytokine comparison in different days of the NEC group (LSD, *P*-value)

Days after modeling	Expression of Bax in each organ						Expression of PCNA in each organ						Expression of PCNA in colon and ileum					
	Day1	Day2	Day3	Day4	Day5	Day6	Day1	Day2	Day3	Day4	Day5	Day6	Day1	Day2	Day3	Day4	Day5	Day6
Day2	0.676						0.035						0.713					
Day3	0.276	0.497					0.003	0.289					0.520	0.778				
Day4	0.099	0.210	0.558				0.000	0.048	0.332				0.031	0.055	0.084			
Day5	0.013	0.033	0.132	0.346			0.000	0.052	0.350	0.971			0.051	0.089	0.137	0.748		
Day6	0.013	0.034	0.134	0.349	0.994		0.000	0.059	0.383	0.920	0.949		0.488	0.737	0.957	0.091	0.149	
Day7	0.002	0.007	0.033	0.110	0.494	0.489	0.000	0.059	0.381	0.923	0.952	0.997	0.139	0.079	0.051	0.003	0.005	0.047
ANOVA	F = 3.154, *P* = 0.017						F = 5.141, *P* = 0.001						F = 4.300, *P* = 0.039					

### Pathological changes of colonic, ileal, lung, liver and kidney injury induced by NEC

On the 1st day after the establishment of the NEC model, we observed intestinal villi falling off, structure disappearance caused by necrosis, submucosal and muscular oedema, intestinal wall congestion, hemorrhage, and necrosis accompanied by infiltration by many inflammatory cells, mainly neutrophils. Histological scores centred at 3 and 4 points on the 1st day, but decreased to 2 points by the 3rd to 5th day, and became < 1 on the 7th day.

In the NEC group, on the 1st day after modeling, pulmonary epithelium, pulmonary interstitial and renal interstitial oedema were accompanied with inflammatory cell infiltration, and inflammatory exudates were seen in the alveolar cavities and bronchi, vacuolar degeneration of hepatocytes, infiltration of inflammatory cells around necrotic foci, ischemic changes of glomeruli and obvious oedema of the proximal convoluted tubule cells. On the 3rd day, alveolar walls continued to thicken, interstitial oedema was prominent, vacuolar degeneration of the liver was alleviated, necrosis of the liver was reduced, the cytoplasm was still loose, glomerular congestion was obvious, and tubular cells were still edematous. On the 5th day, pulmonary oedema and interstitial thickening were significantly alleviated. By the 7th day, alveolar inflammatory exudation and absorption were more visible, hepatic inflammatory cell infiltration was reduced, and glomerular congestion and tubular oedema were significantly alleviated.

### Bax expression in colonic, ileal, lung, liver and kidney

**Fig. 1:**
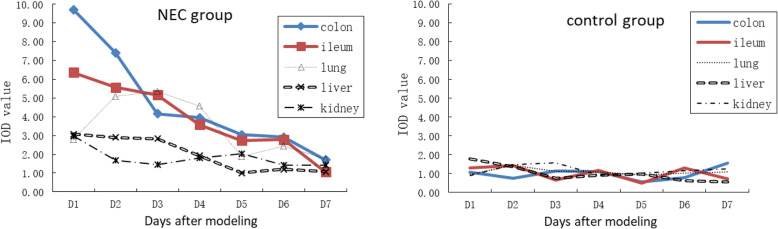
Expression of Bax in the NEC and control groups.

Bax was expressed in intestinal villi epithelial cells, bronchial epithelial cells, inflammatory cells in the pulmonary interstitium and the alveolar area, hepatocytes, renal corpuscles, tubules and medulla. The expression of Bax in the intestinal tract of the NEC group was stronger than that in the control group. Bax expression gradually decreased with time and reached a minimum on the 7th day. The expression of Bax in lung, liver and kidney in the NEC group was higher than that in the control group (*P* < 0.05). Among them, in the lung, the expression of Bax showed a trend of increasing gradually from the 1st day to the 3rd day, and maintained at a high level in the first 5 days. In the liver, the expression of Bax decreased gradually from the 1st day to the 5th day, and then stabilized. In the kidney, the expression of Bax decreased gradually from the 1st day to the 3rd day, then tended to be stable (Fig. 1).

### PCNA expression in colonic, ileal, lung, liver and kidney

**Fig. 2:**
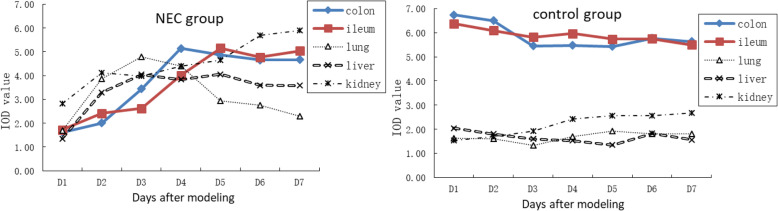
Expression of PCNA in the NEC and control groups.

PCNA is expressed in intestinal epithelial cells, lung epithelium, hepatocytes, interstitial lung, renal cortex and tubular nucleus of the renal corpuscle. In the NEC group, the expression of PCNA on the 1st day after modeling was lower than that of the control group. Expression gradually increased to the 4th and 5th day. Quantitative analysis showed that the expression of PCNA in lung, liver and kidney in the NEC group was higher than that in the control group (*P* < 0.05), except for the lower expression of PCNA in liver on Day 1. The expression of PCNA in the lungs of the NEC group was higher than that of the control group on the 1st day after modeling, and gradually increased to the 3rd day and then decreased to the 7th day. The expression in the liver gradually increased up to Day 3 and remained stable. The expression increased gradually in the kidney (Fig. 2).

### PAF expression in colonic, ileal, lung, liver and kidney

**Fig. 3:**
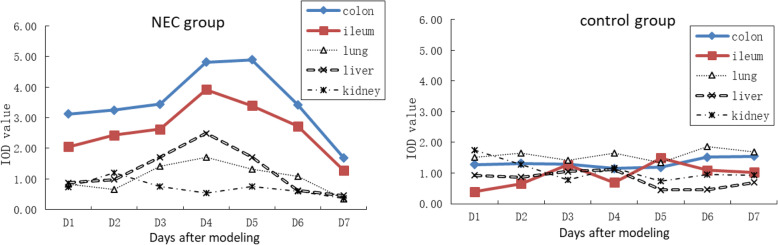
Expression of PAF in the NEC and control groups.

PAF was expressed in intestinal villi epithelial cells, bronchial epithelial cells, inflammatory cells in the lung interstitium and alveolar area, hepatocytes, renal corpuscles, renal tubules and medulla. In the NEC group, expression of PAF was higher than that of the control group on the 1st day, then increased gradually up to the 4th day, after which expression began to decrease and approached normal levels by the 7th day. In the liver, it increased gradually from the 2nd day to the 4th day, and then decreased gradually. However, 1–7 days after the termination of NEC induction, its expression in the kidney was stable. Quantitative analysis showed that the expression of PAF in the lung and liver of the NEC group was higher than that of the control group (*P* < 0.05), but there was no significant difference in the expression of PAF in the kidney (*P* > 0.05) (Fig. 3).

## Discussion

### NEC model establishment and evaluation

Studies have confirmed that the cause of NEC is multifactorial. In recent years, researchers began to try to establish animal models of NEC by the multifactorial combination [9]. In this study, 3-day-old rat pups were used as subjects to establish NEC through artificial feeding, hypoxia and cold stimulation. The results showed that the pathological changes of NEC in neonatal SD rats were obvious, which met the diagnostic criteria of NEC. The NEC rats were consistent with the pathological changes and clinical manifestations of human neonatal NEC [10]. It can therefore be used as a model for NEC.

### Time-series pathological changes of multiple organ injury induced by NEC

Systemic inflammatory response syndrome refers to a kind of uncontrolled systemic inflammatory response caused by various severe infections and non-infectious factors. Further development of systemic inflammatory response syndrome can cause multiple organ dysfunction syndrome [11]. The experimental results showed that the organs of the newborn rats in the NEC group are congested and swollen to varying degrees. On the 1st day after termination of the 3-day NEC induction, inflammatory changes were observed in the intestine, lung, liver and kidney. Unger et al. showed that intestinal flora imbalance can lead to NEC, which leads to systemic inflammatory response syndrome [12]. Therefore, this experiment further confirmed that NEC may lead to an increase in the incidence of multiple organ dysfunction syndrome.

### Significance of apoptosis, proliferation and inflammatory factors in multi-organ in NEC

After induction of NEC, the pathological changes in the intestinal tract, lung, liver and kidney, inflammatory response and repair after injury are accompanied by changes of cell proliferation, apoptosis and inflammatory factors [13]. This may be due to extensive necrosis of typical intestinal tissue at the end of NEC [14]. Apoptosis may be the main mode of death of intestinal epithelial cells in NEC [15]. In the liver and kidney, apoptotic cells induced by secondary injury, caused by inflammatory mediators after NEC, showed different trends as their different reactions. In the lung, the duration of apoptosis may be longer because the lung itself is greatly affected by cold stimulation.

At the end stage, NEC showed extensive necrosis of typical intestinal tissues, but in the early stage, apoptosis may be the main mode of intestinal epithelial cell death. Bax is a major apoptosis gene in inflammatory response related diseases. In this study, the expression of Bax further showed that NEC induced apoptosis in all organs, especially in the most damaged intestine.

At present, PCNA has been widely used in the study of cell proliferation kinetics [16]. In this study, we found that lung, liver and kidney cells proliferated to repair their corresponding organs after injury, but the duration of proliferation and degree of each organ were also different due to their differing abilities to self-repair and mechanism of each organ. In addition, in this study, the intestinal injury was in the positive repair stage only after the 4th day after the model has been established.

In recent years, studies have confirmed that PAF plays a key role in the inflammatory chain reaction of NEC [17]. In this study, the expression of PAF in lung and liver of the NEC group was significantly higher than that of the control group, but there was no change of PAF in the kidney. This may be related to the production of PAF in the kidney and the metabolic level of endogenous PAF. Human beings, like SD rats, produce these cytokines when they are exposed to NEC. Because the above three cytokines are produced in both human and SD rats when NEC occurs, the animal research may be transferred to other species, including human.

In this study, NEC rat model was established by hypoxia and cold stimulation. The damage of liver, kidney and lung caused by hypoxia and cold stimulation may be overlapped with that caused by NEC, which affect the determination of Bax, PCNA and PAF. This is the limitation of the study. We need to determine the damage degree of a single factor on intestinal and extraintestinal tissues in future experiments, so as to exclude the influence of these interference factors.

## Conclusion

In summary, NEC can cause secondary injuries to the colon, ileum, lung, liver and kidney, and the degree and time of injury repair differ. In general, organ repair plays a leading role on the 4th day after modeling.

## Data Availability

The datasets generated and analyzed during the present study are available from the corresponding author on reasonable request.

## Abbreviations

Bax: Bcl2-related X gene
PCNA: Proliferating cell nuclear antigen
PAF: Platelet-activating factor
NEC: Necrotizing enterocolitis
SD: Sprague-dawley
ANOVA: Analysis of variance
LSD: least significant difference
